# Metabolic Characterization of Cerebrospinal Fluid for Patients With Autoimmune Encephalitis: A Preliminary Study

**DOI:** 10.1111/cns.70203

**Published:** 2025-01-03

**Authors:** Xiaolong Li, Xiaoxiao Qin, Yuan Xie, Lingyun Wang, Jinwen Wang, Shushen Ji, Huihui Jiang, Qun Wang

**Affiliations:** ^1^ Department of Neurology, Beijing Tiantan Hospital Capital Medical University Beijing China; ^2^ Department of Neurology, Xiangyang No. 1 People's Hospital Hubei University of Medicine Xiangyang Hubei China; ^3^ Zhangjiang Center for Translational Medicine Shanghai Biotecan Pharmaceuticals co. Ltd. Shanghai China; ^4^ National Center for Clinical Medicine of Neurological Diseases Beijing China; ^5^ Beijing Institute of Brain Disorders, Collaborative Innovation Center for Brain Disorders Capital Medical University Beijing China

**Keywords:** autoimmune encephalitis, biomarker, cerebrospinal fluid, metabolomics, pathophysiology

## Abstract

**Background:**

Metabolomics offers promise in uncovering potential biomarkers and understanding the pathophysiology of autoimmune encephalitis (AE), which is a cluster of disorders with the host immune system targeting self‐antigens expressed in the central nervous system (CNS). In this research, our objective was to explore metabolic characterization in cerebrospinal fluid (CSF) from individuals with AE, aiming to shed light on the pathophysiology of AE.

**Methods:**

A targeted approach was applied using an ultra‐performance liquid chromatography coupled to tandem mass spectrometry (UPLC–MS/MS) system to study CSF metabolites in patients with AE (*n* = 18), and control subjects without neurological diseases (*n* = 17).

**Results:**

A total of 21 potential biomarkers were acquired by getting the intersection of the differential metabolites from univariate statistics and multidimensional statistics between the AE (cell‐based assay panel, CBA‐panel) group and the control group. Specifically, the levels of pyruvic acid and oxoglutaric acid were notably elevated in the AE(CBA‐panel) group compared to those in the control group, indicating that the dysregulated TCA cycle may play a pivotal role in the progression of AE(CBA‐panel). Interestingly, 27 potential biomarkers were acquired by getting the intersection of the differential metabolites from univariate statistics and multidimensional statistics between the anti‐N‐methyl‐D‐aspartate receptor encephalitis (NMDARE) group and the control group, suggesting that the disparities between patients with greater homogeneity and the controls are amplified. In addition, seven differential metabolites were identified by the univariate statistics between the AE (tissue‐based assay, TBA) group and the control group, including alpha‐linolenic acid and gamma‐linolenic acid, suggesting that dysregulated biosynthesis of unsaturated fatty acids and alpha‐linolenic acid metabolism might be crucial in the AE(TBA) disease course.

**Conclusion:**

Collectively, distinct metabolic profiles were evident in the CSF of the AE group compared to the control group, notably involving metabolites associated with mitochondrial dysfunction, which helped to elucidate the pathophysiology of AE.

## Graphical abstract

Compared to control subjects without neurological diseases, distinct metabolic profiles were evident in the cerebrospinal fluid of patients with autoimmune encephalitis (AE) diagnosed by fluorescence pattern in both cell‐based assays and tissue‐based assays, which helped to elucidate the pathophysiology of AE.

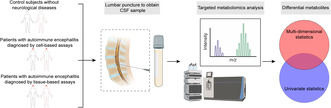

## 1 Introduction

Autoimmune encephalitis (AE) encompasses a range of conditions wherein the body's immune system directs its attack toward self‐antigens present within the central nervous system (CNS) [1]. Some of the most well‐defined conditions are linked to autoantibodies that target neuroglial antigens. These autoantibodies are classified as pathogenic due to their targeting of the extracellular domains of their target antigens [2, 3, 4]. Numerous recognized antigens comprise vital synaptic proteins, ion channels, or receptors, indicating that autoantibodies directed at the extracellular domain are likely to influence crucial physiological functions actively.

While comprehensive, randomized, double‐blinded trials investigating immune modulation in AE remain scarce, a growing body of evidence and current guidelines suggest the advantages of initiating treatment promptly [5, 6, 7, 8]. Certainly, the efficacy of immune therapies in AE might be diminished in instances where there are delays in diagnosis and treatment [9], underscoring the critical necessity for prompt identification. Delays in diagnosis have been identified as a significant contributor to extended hospital stays, thereby escalating healthcare expenses [10]. The persistence of such delays may stem from ongoing insufficient recognition of AE syndromes and delays associated with access to testing resources. Indeed, cerebrospinal fluid (CSF) examinations to detect antibodies against neuronal cell surface or synaptic proteins are generally not readily accessible in the majority of hospitals, necessitating the dispatch of samples to specialized laboratories for analysis, resulting in turnaround times ranging from 5 to 10 days.

CSF has been regarded as a “liquid biopsy” for CNS pathology since the advent of lumbar puncture in the nineteenth century. Measuring metabolites in CSF holds great promise as a strategy to reveal pathogenesis for infectious and inflammatory disorders of CNS, given that CSF intimately interacts with the target organ [11, 12, 13]. Additionally, functional small molecule metabolites hold promise for auxiliary diagnostic biomarkers that can be utilized bedside or in acute care laboratories following lumbar puncture. This has the potential to offer faster results compared to the detection of pathogenic autoantibodies. Earlier studies have documented the discovery of precise biomarkers for various forms of encephalitis, such as enteroviral meningitis [14], varicella zoster virus meningitis/encephalitis [15], anti‐N‐methyl‐D‐aspartate receptor encephalitis (NMDARE) [16, 17]. So far, the concentrations of kynurenine (Kyn) and tryptophan (Trp) in CSF samples have drawn a lot of interest for autoimmune neuroinflammatory diseases [17, 18, 19]. Luo et al. reported that Kyn concentrations, Trp concentrations, and Kyn/Trp ratios in patients with NMDARE all were similar to those in controls [17]. However, the majority of control groups in the aforementioned publications consisted of individuals with noninflammatory neurological diseases (such as normal pressure hydrocephalus, idiopathic intracranial hypertension, Tourette syndrome, or Bell's palsy). There is a paucity of studies where the control group closely matches the health status of individuals without any neurological disorders. Moreover, there has been no utilization of a focused metabolomics strategy encompassing over 300 small molecules to analyze CSF metabolites in patients with NMDARE.

To assist physicians in promptly considering the possibility of AE, we have expanded the metabolic characteristics of CSF in patients with AE through a targeted metabolomics approach with 310 small molecules. Moreover, we also aimed to investigate the differing metabolic pathways between individuals with AE and controls, to gain a deeper understanding of the disease's pathophysiology.

## 2 Materials and Methods

### 2.1 Study Cohort

Thirty‐five patients were recruited at the Xiangyang No. 1 People's Hospital, Hubei University of Medicine (Xiangyang, China) from May 2022 to November 2023. This study was approved by the Ethics Committee of the Xiangyang No. 1 People's Hospital, Hubei University of Medicine. For patients with AE (*n* = 18), 12 patients were diagnosed by fluorescence pattern in cell‐based assays (AE(CBA‐panel), NMDAR‐positive cases *n* = 5, contactin‐associated protein‐like 2 (CASPR2)‐positive cases *n* = 1, leucine‐rich glioma‐inactivated 1 (LGI1)‐positive cases *n* = 2, glutamic acid decarboxylase‐65 (GAD65)‐positive cases *n* = 1, gamma‐aminobutyric acid B receptor (GABABR)‐positive cases *n* = 1, myelin oligodendrocyte glycoprotein (MOG)‐positive cases *n* = 1, and Kelch‐like protein 11 (KLHL11)‐positive cases *n* = 1), and six patients were identified by tissue‐based assays (AE(TBA)). The clinical presentation of AE cases varied but typically included symptoms such as psychiatric symptoms (*n* = 14), memory disturbances (*n* = 7), lalopathy (*n* = 8), seizures (*n* = 10), and dyskinesia (*n* = 9) (Table S1). The diversity of symptoms is consistent with the involvement of different brain regions and reflects the heterogeneity of AE presentations. For the control group (*n* = 17), all patients were non‐neurological diseases, consisting of fracture (*n* = 4), joint replacement (*n* = 3), mixed hemorrhoid (*n* = 5), varicosity (*n* = 1), inguinal hernia (*n* = 1), anal fistula (*n* = 2), and vaginal prolapse (*n* = 1). Before inclusion, all patients were thoroughly informed about the purpose of the study and the nature of the sample collection. Written informed consent was obtained from each patient. All CSF samples in the control group were obtained during spinal anesthesia. In the case of AE patients, 13 CSF samples were collected during the acute phase and before the initiation of immune modulatory therapy (corticosteroids or intravenous immunoglobulin). However, four patients had been treated with immune modulatory therapy, and one patient had received antiepileptic therapy (levetiracetam tablets). The average time from CSF collection to initial processing was approximately 15 min, with a range of 10 to 20 min. After collection, the CSF samples were immediately aliquoted into prechilled cryotubes and snap‐frozen in liquid nitrogen to preserve their metabolic integrity. Then, the samples were transferred to a −80°C freezer, and the average duration of storage before analysis was approximately 8 months, with a range of 2 to 15 months. Lastly, CSF samples were shipped to the testing laboratory using dry ice to maintain a temperature of approximately −78.5°C during transport. The average shipping time was 24 h, with a maximum duration of 48 h. All samples were monitored to ensure that they remained frozen throughout the shipping process. Table 1 displays the standard blood and CSF parameters.

**TABLE 1:** Demographic and clinical laboratory characteristics.

Variable	Control	AE(CBA‐panel)	NMDARE	AE(TBA)	*p* ^a^		
					Control vs. AE(CBA‐panel)	Control vs. NMDARE	Control vs. AE(TBA)
Total, no.	17	12	5	6			
Demographic parameter							
Sex, no.							
Female	9	6	2	3	1.0000^b^	1.0000^b^	1.0000^b^
Male	8	6	3	3			
Smoking, no.							
Yes	5	2	1	0	0.6645^b^	1.0000^b^	0.2725^b^
No	12	10	4	6			
Drinking, no.							
Yes	3	2	1	0	1.0000^b^	1.0000^b^	0.5392^b^
No	14	10	4	6			
Diabetes, no.							
Yes	2	1	0	0	1.0000^b^	1.0000^b^	1.0000^b^
No	15	11	5	6			
Hypertension, no.							
Yes	1	3	2	0	0.2785^b^	0.1169^b^	1.0000^b^
No	16	9	3	6			
Age (y), median (range)	57.00 (26.00–78.00)	38.50 (17.00–80.00)	41.00 (19.00–66.00)	27.00 (8.00–54.00)	0.0414	0.1842	0.0115
Height (cm), median (range)	167.00 (155.00–180.00)	167.50 (160.00–185.00)	175.00 (165.00–175.00)	169.00 (100.00–173.00)	0.2113	0.0351	0.5755
Weight (kg), median (range)	64.00 (51.00–80.00)	67.00 (55.00–80.00)	75.00 (60.00–77.00)	59.50 (25.00–80.00)	0.1821	0.1144	0.8251
Blood parameter, median (range)							
Leukocyte count (*10^9^/L)	5.020 (2.990–10.910)	7.680 (3.830–18.520)	12.530 (7.610–15.300)	6.145 (3.880–20.250)	0.0189	0.0032	0.6078
Neutrophile granulocyte	3.000 (1.290–9.210)	6.115 (2.720–14.720)	10.440 (4.430–12.580)	3.665 (1.470–16.970)	0.0030	0.0030	0.6474
Lymphocyte	1.600 (0.090–3.370)	1.440 (0.490–2.540)	1.560 (0.490–2.540)	2.040 (1.510–2.420)	0.7853	0.8345	0.1129
Neutrophil‐to‐lymphocyte ratio (NLR)	1.610 (0.950–10.230)	6.215 (1.660–12.930)	6.990 (1.740–12.930)	1.900 (0.720–7.280)	0.0036	0.0238	0.9072
Total protein (TP, g/L)	69.70 (54.90–76.60)	66.65 (52.90–82.80)	67.30 (52.90–82.80)	64.46 (62.10–73.10)	0.0958	0.3347	0.2151
Albumin (alb, g/L)	41.20 (32.30–49.30)	34.85 (29.20–43.90)	35.30 (34.10–43.90)	39.20 (37.20–46.40)	0.0005	0.0825	0.3470
Bilirubin	15.30 (8.10–71.80)	15.25 (12.10–31.70)	13.30 (12.10–16.40)	12.40 (8.60–17.00)	0.6391	0.1964	0.0544
Serum creatinine (SCR)	64.02 (40.45–78.30)	66.80 (39.00–441.50)	75.90 (41.10–441.50)	55.30 (32.90–84.60)	0.8040	0.2827	0.5335
C‐reactive protein (CRP)	1.000 (0.450–2.500)	31.06 (0.100–185.50)	25.06 (0.100–104.000)	6.600 (5.500–8.400)	< 0.0001	0.0450	< 0.0001
Procalcitonin (PCT)	0.050 (0.050–0.050)	0.310 (0.040–2.260)	0.110 (0.040–0.3000)	0.055 (0.050–0.070)	< 0.0001	0.0058	0.0113
Interleukin‐6 (IL‐6)	2.200 (1.500–63.60)	27.35 (12.20–135.60)	13.50 (12.20–25.10)	12.70 (0.100–25.60)	< 0.0001	0.0009	0.1110
CSF parameter, median (range)							
CSF pressure	100.00 (80.00–120.00)	155.00 (80.00–302.00)	170.00 (130.00–302.00)	130.00 (110.00–150.00)	0.0011	< 0.0001	0.0025
Leukocyte count (*10^6^/L)	0.00 (0.00–2.00)	17.50 (4.00–50.00)	20.00 (10.00–34.00)	1.50 (1.00–12.00)	< 0.0001	< 0.0001	0.0021
CSF‐TP (g/L)	0.1100 (0.0800–0.2000)	0.4200 (0.1500–1.1700)	0.2698 (0.1500–0.4006)	0.2990 (0.1324–0.5956)	< 0.0001	0.0015	0.0004
Glucose (mmol/L)	3.200 (2.300–4.200)	4.055 (0.410–7.060)	3.710 (0.410–4.370)	3.410 (3.050–3.990)	0.0944	0.8035	0.7444
Chlorides (mmol/L)	120.50 (116.50–132.60)	122.30 (110.00–131.30)	118.40 (110.00–130.20)	121.50 (116.90–126.90)	0.8873	0.3278	0.6969

*Note:* Data are median values with their respective ranges, unless otherwise indicated.

^a^ By the Mann–Whitney *U* test, unless otherwise indicated. *p* < 0.05 are statistically significant.

^b^ By the fisher's exact test.

### 2.2 Targeted Metabolomics

Targeted liquid chromatography (LC) with tandem mass spectrometry (MS) analysis was conducted employing an UPLC–MS/MS system (ACQUITY UPLC‐Xevo TQ‐S). A collective of 310 standard substances was sourced from Sigma‐Aldrich (St. Louis, MO, USA), Steraloids Inc. (Newport, RI, USA), and TRC Chemicals (Toronto, ON, Canada). Each of the standard substances underwent precise weighing and was dissolved in a suitable solvent to produce individual stock solutions at a concentration of 5.0 mg/mL. A suitable quantity of each stock solution was combined to generate stock calibration solutions.

CSF samples were thawed using an ice bath to minimize sample degradation. Two hundred microliters of CSF was pipetted into a 96‐well plate and subjected to lyophilization (Labconco, Kansas City, MO, USA). The dried samples were then reconstituted with 20 μL of 50% methanol. Subsequently, the plate was transferred to the Eppendorf epMotion Workstation (Eppendorf Inc., Humburg, Germany). One hundred and twenty microliters of ice‐cold methanol containing internal standards was automatically dispensed into each sample well and vortexed for 5 min. The samples were then centrifuged at 4000 × *g* for 30 min using the Allegra X‐15R centrifuge (Beckman Coulter Inc., Indianapolis, IN, USA). Following centrifugation, 30 μL of the supernatant was transferred to a clean 96‐well plate, and 20 μL of freshly prepared derivative reagents were added to each well for derivatization, which was carried out at 30°C for 60 min. After derivatization, 330 μL of ice‐cold 50% methanol solution was added to dilute the samples. The plate was then stored at −20°C for 20 min and subsequently centrifuged at 4000 g for 30 min at 4°C. Finally, the supernatant was collected for LC‐MS analysis.

The mobile phases comprised 0.1% formic acid in water (mobile phase A) and a mixture of acetonitrile/isopropanol (70:30, mobile phase B). Each sample (5 μL) was injected into an ACQUITY UPLC BEH C18 VanGuard precolumn (1.7 μm, 2.1 × 5 mm) and an ACQUITY UPLC BEH C18 analytical column (1.7 μm, 2.1 × 100 mm) (Waters), maintained at 40°C. The flow rate was set at 0.40 mL/min, following the mobile‐phase gradient as follows: 0.0–1.0 min (5% B), 1.0–11.0 min (5%–78% B), 11.0–13.5 min (78%–95% B), 13.5–14.0 min (95%–100% B), 14.0–16.0 min (100% B), 16.0–16.1 min (100%–5% B), and 16.1–18.0 min (5% B). The mass spectrometer was operated in negative mode with a 2.0‐kV capillary voltage and in positive mode with a 1.5‐kV capillary voltage. Source and desolvation temperatures were set at 150°C and 550°C, respectively.

The raw data targeted for analysis underwent processing via a calibration curve of standards. The calibration curves were assessed based on instrument response and standard concentration. Subsequently, the targeted metabolites were analyzed using iMAP software (version 1.0; Metabo‐Profile, Shanghai, China). All data underwent analysis using R (version 3.5.0), and differential metabolomic analysis was conducted utilizing the MSstats R package. This process involved log2 transformation, normalization, and *p*‐value calculation on the spectronaut and skyline quantitative data. The hierarchical cluster analysis was executed using the hcaMethods R package and the distance calculation employed the Euclidean distance with the heatmap R package, which aimed to uncover the correlation between samples and metabolites. The normality of the data distribution was assessed using the Shapiro–Wilk test. For statistical comparisons, normally distributed metabolites were analyzed using a two‐tailed student's *t*‐test, while the Mann–Whitney *U*‐test was applied for non‐normally distributed metabolites. Alongside the univariate statistics, orthogonal partial least square discriminant analysis (OPLS‐DA) was also employed to maximize the identification of metabolic profile differences between groups. Metabolites with a *p*‐value < 0.05 in univariate statistics and a variable importance in projection (VIP) score ≥ 1 in multidimensional statistics were considered statistically significant. The enrichment of Kyoto encyclopedia of genes and genomes (KEGG) signaling pathways was performed utilizing the KEGG database (version 89.1).

### 2.3 Statistical Analysis

Data were analyzed with SPSS 23.0 (IBM, Chicago, IL, United States) and GraphPad Prism version 7.0 (GraphPad, San Diego, CA, United States). For categorical variables, fisher's exact test was used to assess the difference between the two groups. For continuous variables, the Shapiro–Wilk normality test was used to assess data distribution. Statistical comparisons between the two groups were conducted using a two‐tailed Student's *t*‐test for normally distributed data and a Mann–Whitney *U*‐test for non‐normally distributed data. Exact numbers of patients and values of differential metabolites for each group are reported in the figure legends. In all cases, a *p*‐value < 0.05 was considered statistically significant.

## 3 Results

### 3.1 Study Cohort

Demographic and clinical data of the control group, AE(CBA‐panel) group, NMDARE group, and AE(TBA) group are summarized in Table 1. Both the AE(CBA‐panel) and the AE(TBA) groups exhibited a younger age profile compared to the control group, which was consistent with the epidemiology of AE. For the blood parameter, the levels of neutrophile granulocyte, neutrophil‐to‐lymphocyte ratio (NLR), C‐reactive protein (CRP), procalcitonin (PCT), interleukin‐6 (IL‐6) in the AE(CBA‐panel) group were much higher than these in the control group. However, only CRP and PCT exhibited significantly increased levels in the AE(TBA) group compared to the control group. In terms of CSF parameters, both the AE(CBA‐panel) and AE(TBA) groups demonstrated significantly increased levels of CSF pressure, leukocyte count, and CSF‐TP in comparison to the control group. In addition, the trends of the above blood and CSF parameters in the NMDARE group were consistent with those in the AE(CBA‐panel) group. Lastly, the control group exhibited normal values for all standard parameters outlined in Table 1.

### 3.2 CSF Metabolite Populations Differ Between the AE(CBA‐Panel) Group and the Control Group

**FIGURE 1:**
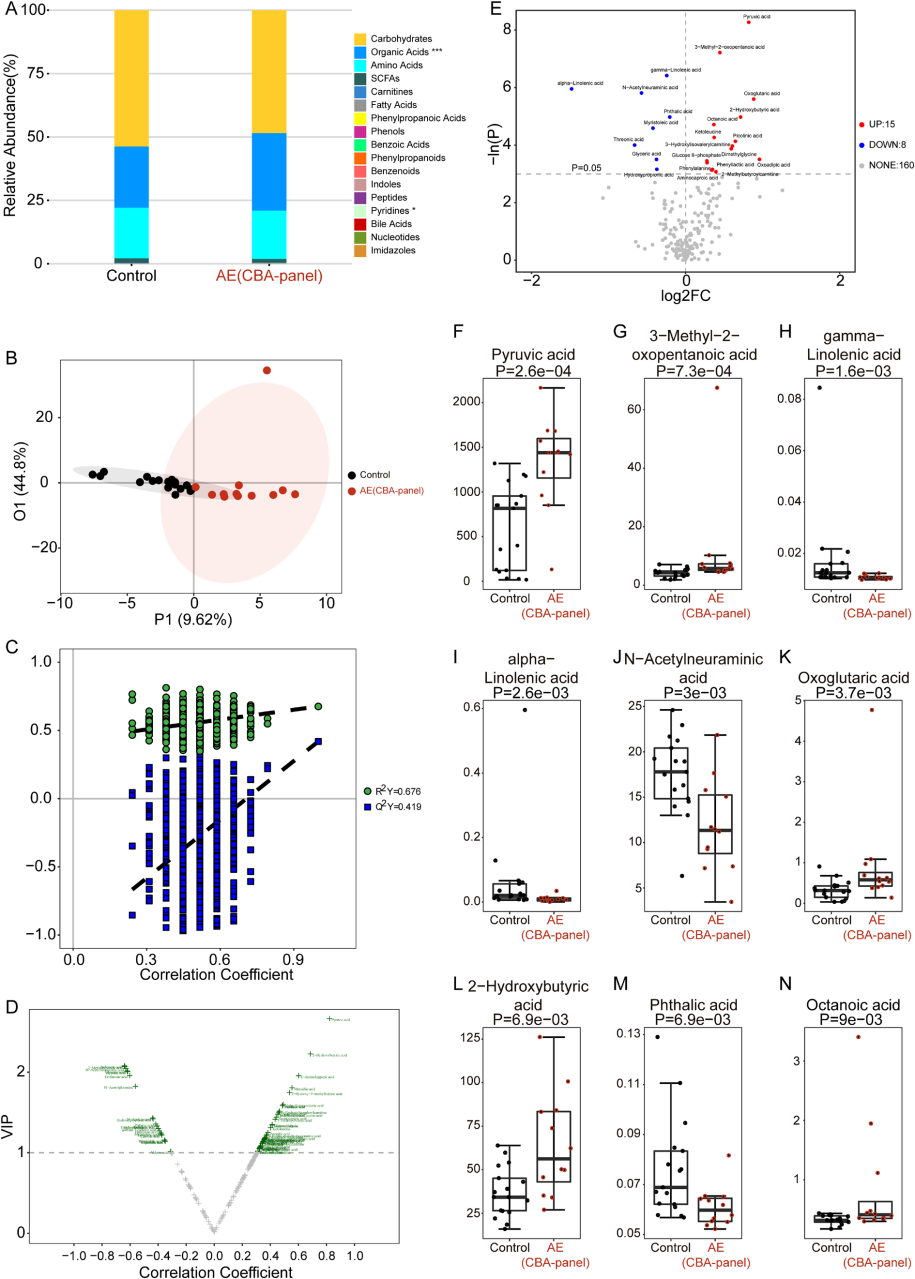
Global differences of CSF metabolites between the control group (*n* = 17) and the AE(CBA‐panel) group (*n* = 12). (A) The relative abundance of each metabolite class in the control group and the AE(CBA‐panel) group is shown in a stacked bar chart. OPLS‐DA 2D score plot (B), permutation test result (C), and volcano plot of OPLS‐DA model (D) helped to select differential metabolites based on multidimensional Statistics. (E) The volcano plot of univariate statistics highlighted specific metabolite differences discerned through univariate statistical analysis conducted between the control group and the AE(CBA‐panel) group. Relative to the control group, metabolites exhibiting distinctive changes in the upper right quadrant (highlighted in red) and in the upper left quadrant (highlighted in blue) are notably elevated and reduced, respectively, within the AE(CBA‐panel) group. Boxplot of top nine differential metabolites between groups by the univariate statistical analysis is ordered by *p* value, including pyruvic acid (F), 3‐Methyl‐2‐oxopentanoic acid (G), gamma‐linolenic acid (H), alpha‐linolenic acid (I), N‐Acetylneuraminic acid (J), oxoglutaric acid (K), 2‐Hydroxybutyric acid (L), phthalic acid (M), and octanoic acid (N). Values are presented as medians with interquartile ranges (IQR).

**FIGURE 2:**
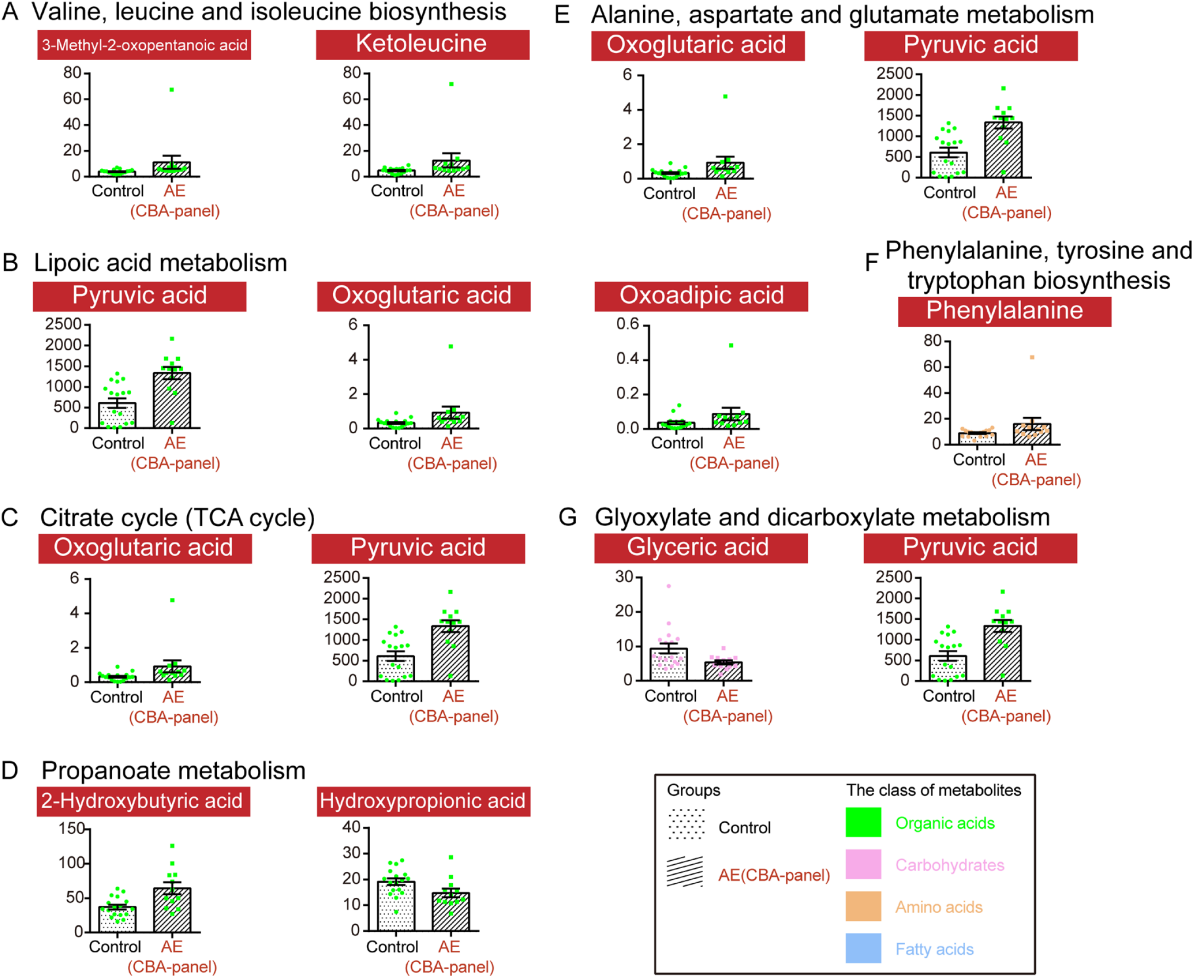
Disordered signaling pathways were enriched by the small molecule pathway database and KEGG analysis based on the differentially expressed metabolites between the control group (*n* = 17) and the AE(CBA‐panel) group (*n* = 12), including valine, leucine and isoleucine biosynthesis (A), lipoic acid metabolism (B), citrate cycle (TCA cycle) (C), propanoate metabolism (D), alanine, aspartate, and glutamate metabolism (E), phenylalanine, tyrosine, and tryptophan biosynthesis (F), and glyoxylate and dicarboxylate metabolism (G). Each red box in a pathway represented a metabolite. Bar plots display the mean absolute concentrations of metabolites with standard error of the mean (SEM) for the control and AE(CBA‐panel) groups.

To quantify the changes in circulating metabolites between the AE(CBA‐panel) group and the control group, we performed the targeted metabolomics analysis using a Q300 Kit (Metabo‐Profile, Shanghai, China). From the concentrations observed in various groups, the 201 metabolites were categorized into 17 distinct biochemical classes, including carbohydrates, organic acids, amino acids, SCFAs, carnitines, fatty acids, phenylpropanoic acids, phenols, benzoic acids, phenylpropanoids, benzenoids, indoles, peptides, pyridines, bile acids, nucleotides, and imidazoles (Figure 1A). We conducted a comparison of the metabolomic profiles between the AE(CBA‐panel) group and the control group to pinpoint variances in metabolite levels through the multidimensional statistics and the univariate statistics. Although no significant difference was found for these two groups in the OPLS‐DA model (Figure 1B), 72 differential metabolites were identified based on correlation coefficient and VIP data (Figure 1C,D). For the univariate statistical analysis, compared with the control group, 15 metabolites with red highlights and eight metabolites with blue highlights were significantly increased and decreased in the AE(CBA‐panel) group, respectively (Figure 1E). Pyruvic acid, 3‐methyl‐2‐oxopentanoic acid, gamma‐linolenic acid, alpha‐linolenic acid, N‐acetylneuraminic acid, oxoglutaric acid, 2‐hydroxybutyric acid, phthalic acid, and octanoic acid were the nine top differential metabolites, whose values were presented as medians with interquartile ranges (IQR) (Figure 1F–N). In addition, we also explored the abnormal signaling pathways between these two groups based on 23 potential biomarkers, which were filtered by the following filter conditions: (1) VIP > 1 in the multidimensional statistics; and (2) *p* < 0.05 in the univariate statistics (Figure S1). Disordered pathways were enriched by the small molecule pathway database and KEGG analysis, which includes valine, leucine and isoleucine biosynthesis, lipoic acid metabolism, citrate cycle (TCA cycle), propanoate metabolism, alanine, aspartate and glutamate metabolism, phenylalanine, tyrosine and tryptophan biosynthesis, and glyoxylate and dicarboxylate metabolism (Figure S2 and Figure 2). In particular, pyruvic acid and oxoglutaric acid, both of which are crucial components in the above‐disordered pathways, presented with a significant increase in the AE(CBA‐panel) group compared to the control group (Figure 2).

### 3.3 Targeted Metabolomic Analysis Revealed Differential Metabolites and Metabolic Pathways Between the NMDARE Group and the Control Group

**FIGURE 3:**
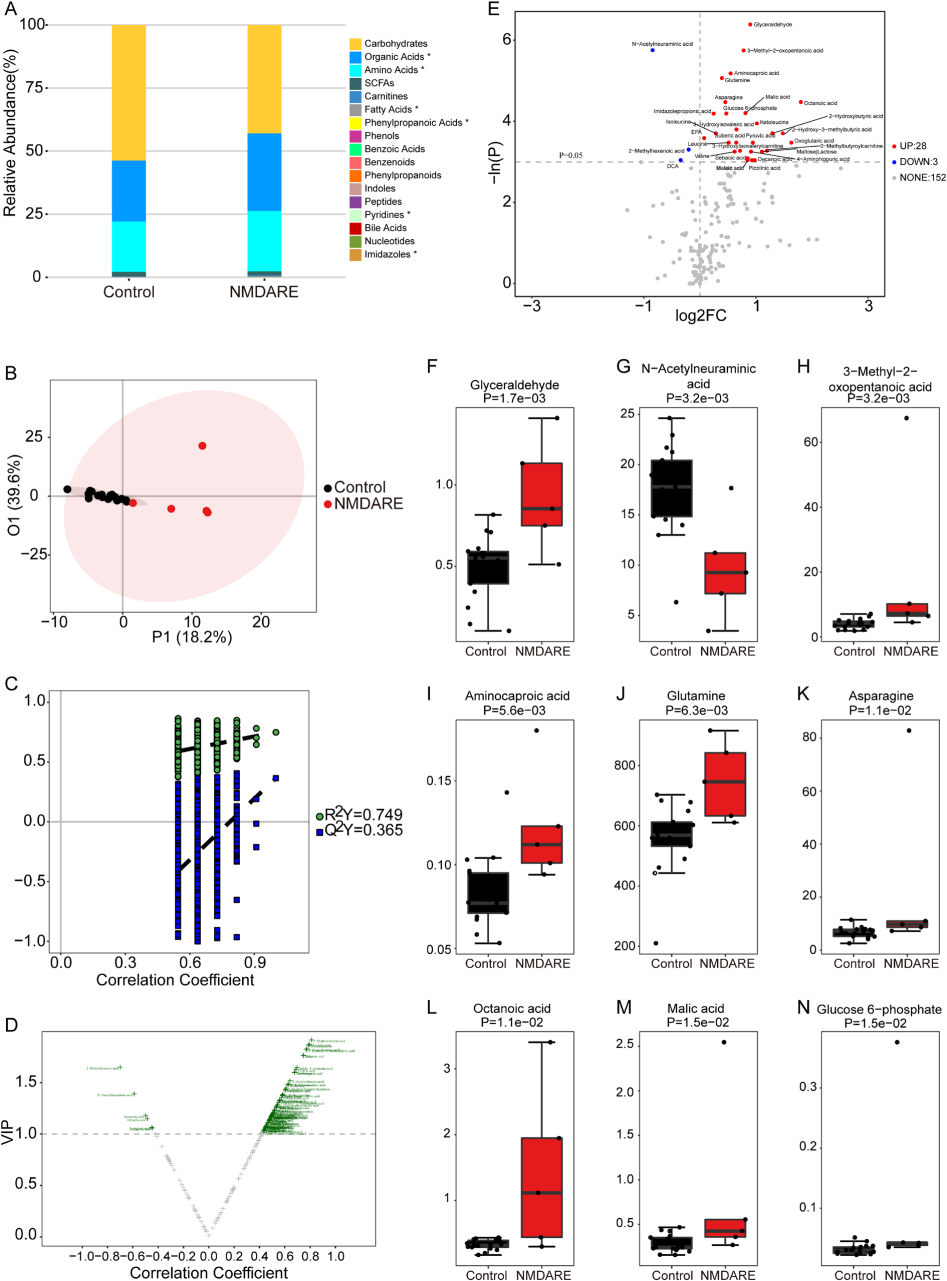
Significant variations in CSF metabolites were observed between the control group (*n* = 17) and the NMDARE group (*n* = 5). (A) A stacked bar chart illustrates the proportional representation of metabolite classes in both the control group and the NMDARE group. The OPLS‐DA 2D score plot (B), permutation test result (C), and the volcano plot derived from the OPLS‐DA model (D) were used to identify differentially expressed metabolites through multidimensional statistics. (E) The volcano plot derived from univariate statistical analysis revealed distinct metabolite differences between the control group and the NMDARE group. In contrast to the control group, metabolites showing red highlights in the upper right corner and blue highlights in the upper left corner are markedly elevated and reduced, respectively, in the NMDARE group. The boxplot depicting the top nine differentially expressed metabolites between groups, identified through univariate statistical analysis, is arranged according to the significance level indicated by the *p* value. These metabolites include glyceraldehyde (F), N‐Acetylneuraminic acid (G), 3‐Methyl‐2‐oxopentanoic acid (H), aminocaproic acid (I), glutamine (J), asparagine (K), octanoic acid (L), malic acid (M), and glucose 6‐phosphate (N). Values are presented as medians with IQR.

**FIGURE 4:**
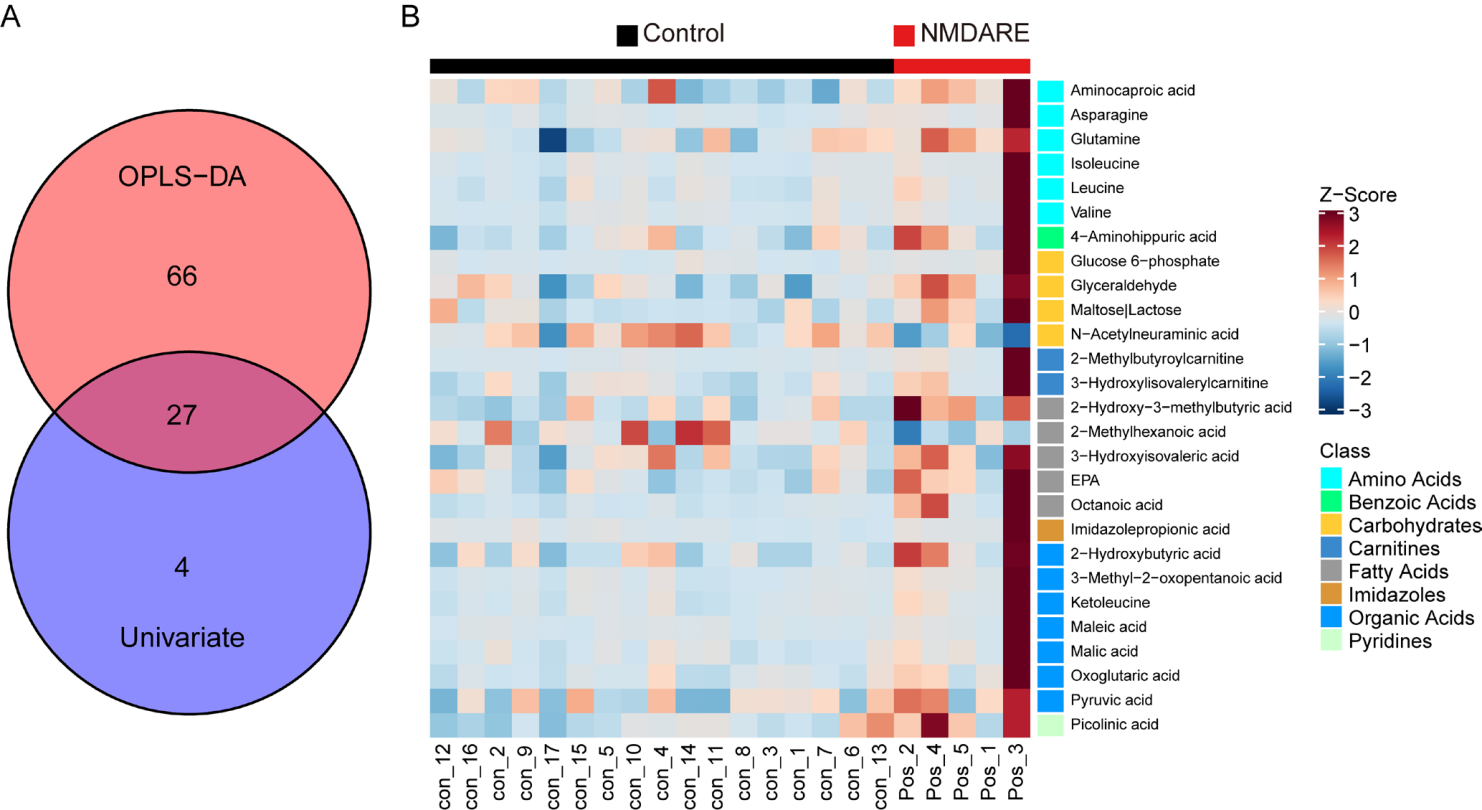
(A) A Venn plot of differential metabolites identified through multidimensional statistics and univariate statistics is presented for both the control group (*n* = 17) and the NMDARE group (*n* = 5). Differential metabolites within the intersection set undergo filtering by VIP > 1 in multidimensional statistics and by criteria of *p* < 0.05 in univariate statistics. (B) The heatmap displaying the differential metabolites is organized according to class, with the names of each sample from both groups.

**FIGURE 5:**
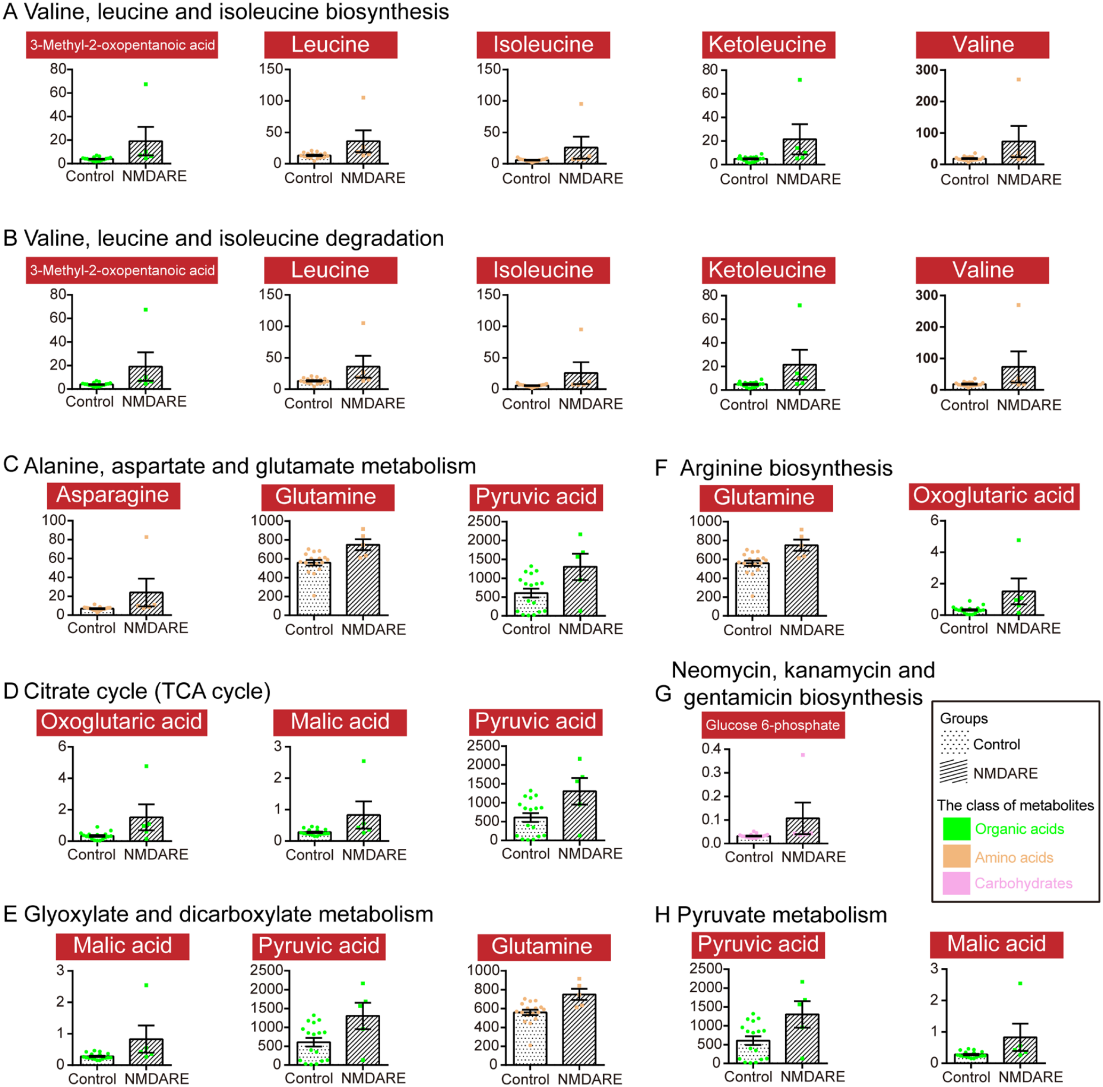
Disordered signaling pathways were enriched by the small molecule pathway database and KEGG analysis based on the differential metabolites between the control group (*n* = 17) and the NMDARE group (*n* = 5), including valine, leucine and isoleucine biosynthesis (A), valine, leucine and isoleucine degradation (B), alanine, aspartate, and glutamate metabolism (C), citrate cycle (TCA cycle) (D), glyoxylate and dicarboxylate metabolism (E), arginine biosynthesis (F), neomycin, kanamycin, and gentamicin biosynthesis (G), and pyruvate metabolism (H). In each pathway, red boxes denote individual metabolites. Bar plots display the mean absolute concentrations of metabolites with SEM for the control and NMDARE groups.

To characterize unique metabolites and metabolic pathways associated with NMDARE, we conducted a comparison of metabolomic profiles between the NMDARE group and the control group. Utilizing the concentrations observed in these two groups, we categorized 201 metabolites into 17 distinct biochemical classes (Figure 3A). We adopted multidimensional statistics and univariate statistics to identify differential metabolites between the two groups. While the OPLS‐DA model did not reveal a significantly distinct contrast (Figure 3B), a total of 104 differential metabolites were discerned using correlation coefficient and VIP data (Figure 3C,D). In the univariate statistical analysis, when compared to the control group, 28 metabolites were significantly elevated (highlighted in red), while three metabolites exhibited significant reductions (highlighted in blue) in the NMDARE group (Figure 3E). Glyceraldehyde, N‐Acetylneuraminic acid, 3‐Methyl‐2‐oxopentanoic acid, aminocaproic acid, glutamine, asparagine, octanoic acid, malic acid, and glucose 6‐phosphate were the top nine differential metabolites, which were presented as medians with IQR in the Figure 3F–N, respectively. Moreover, we investigated the varying metabolic pathways between these two groups using 27 potential biomarkers, which were selected based on the following criteria: (1) VIP > 1 in the multidimensional statistics; and (2) *p* < 0.05 in the univariate statistics (Figure 4). Pathway analysis by the small molecule pathway database and KEGG showed that valine, leucine and isoleucine biosynthesis, valine, leucine and isoleucine degradation, alanine, aspartate and glutamate metabolism, citrate cycle (TCA cycle), glyoxylate and dicarboxylate metabolism, arginine biosynthesis, neomycin, kanamycin and gentamicin biosynthesis, and pyruvate metabolism were disordered (Figure S3 and Figure 5). Specifically, all metabolites implicated in the aforementioned eight disrupted pathways exhibited a notable increase in the NMDARE group compared to the control group (Figure 5).

### 3.4 CSF Metabolite Populations Differ Between the AE(TBA) Group and the Control Group

**FIGURE 6:**
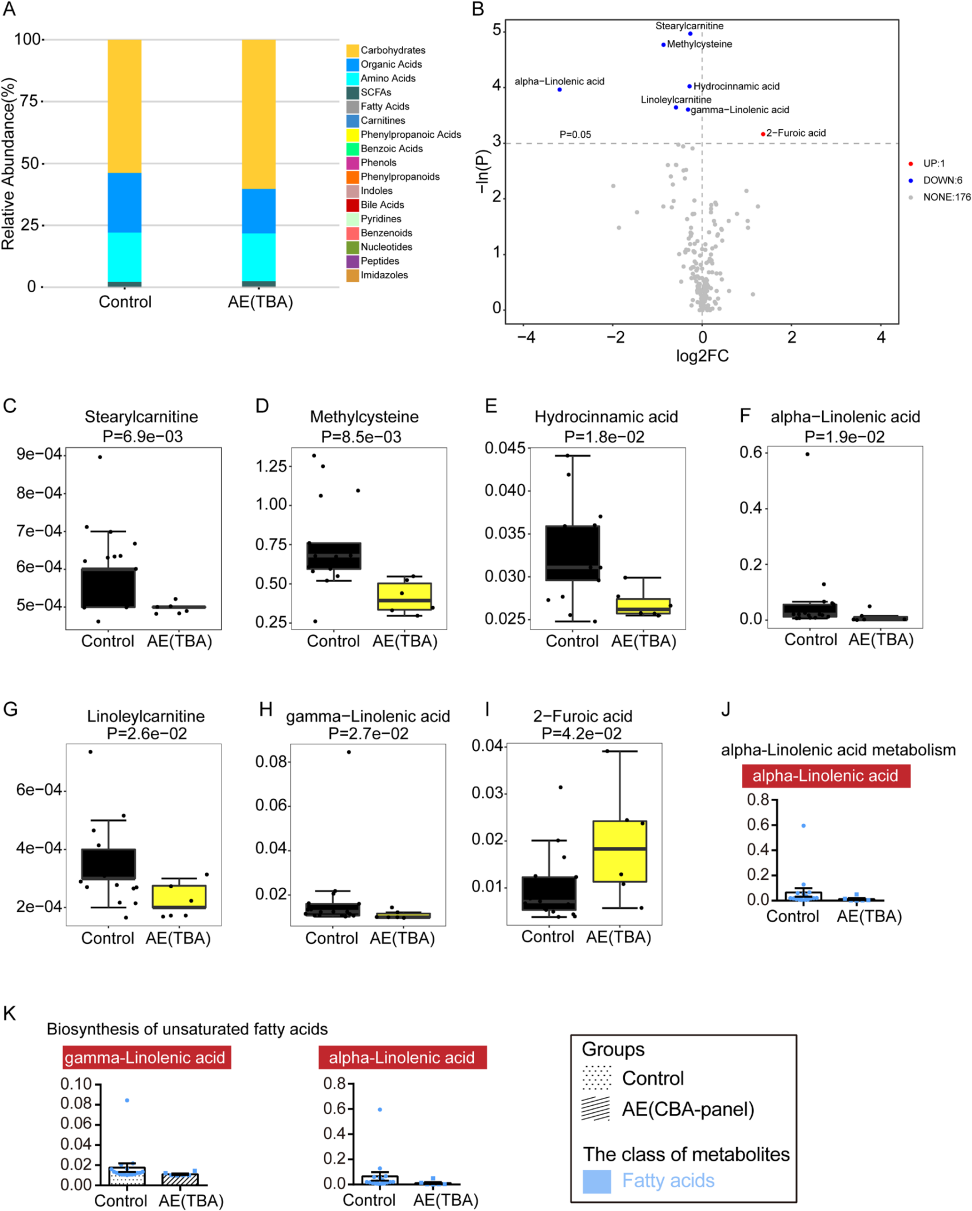
Global differences of CSF metabolites between the control group (*n* = 17) and the AE(TBA) group (*n* = 6). (A) The relative abundance of each metabolite class in the control group and the AE(TBA) group is shown in a stacked bar chart. The volcano plot of univariate statistics (B) indicated the differential metabolites obtained by univariate statistical analysis between the control group and the AE(TBA) group. Compared with the control group, differential metabolites (points with red highlight) in the right top corner and differential metabolites (points with blue highlight) in the left top corner are significantly increased and decreased in the AE(TBA) group, respectively. Boxplot of seven differential metabolites between these two groups by the univariate statistical analysis is ordered by *p* value, including stearylcarnitine (C), methylcysteine (D), hydrocinnamic acid (E), alpha‐linolenic acid (F), linoleylcarnitine (G), gamma‐linolenic acid (H), and 2‐Furoic acid (I). Values are presented as medians with IQR. Disordered signaling pathways were enriched by the Small Molecule Pathway Database and KEGG analysis based on the differential metabolites filtering by *p* < 0.05 in univariate statistics between these two groups, including alpha‐linolenic acid metabolism (J) and biosynthesis of unsaturated fatty acids (K). Each red box in a pathway represented a metabolite. Bar plots display the mean absolute concentrations of metabolites with SEM for the Control and AE(TBA) groups.

To identify particular metabolites and metabolic pathways associated with AE confirmed by tissue‐based assay, we conducted a comparison of metabolomic profiles between the AE(TBA) group and the control group. Using the concentrations detected in these two groups, a total of 201 metabolites were classified into 17 distinct biochemical categories (Figure 6A). As the OPLS‐DA model construction failed, only seven differential metabolites were acquired by the univariate statistics between the two groups (Figure 6B). Compared with the control group, stearylcarnitine, methylcysteine, hydrocinnamic acid, alpha‐linolenic acid, linoleylcarnitine, and gamma‐linolenic acid were significantly decreased in the AE(TBA) group (Figure 6C–H), while only 2‐furoic acid showed a notable increase (Figure 6I). Pathway analysis using the small molecule pathway database and KEGG revealed disturbances in both alpha‐linolenic acid metabolism and biosynthesis of unsaturated fatty acids (Figure 6J,K).

## 4 Discussion

In recent times, there have been several targeted CSF metabolomics investigations carried out on cohorts with AE, utilizing the combination of liquid chromatography and high‐resolution mass spectrometry [15]. This study systematically provides a comprehensive view of the metabolic characterization of CSF from AE patients using a targeted metabolomics approach, especially adopting control subjects without neurological diseases. A total of 21 potential biomarkers were acquired by getting the intersection of the differential metabolites from univariate statistics and multidimensional statistics between the AE(CBA‐panel) group and the control group. Specifically, the levels of pyruvic acid and oxoglutaric acid were notably elevated in the AE(CBA‐panel) group compared to those in the control group, indicating that the dysregulated TCA cycle may play a pivotal role in the progression of AE(CBA‐panel). Interestingly, 27 potential biomarkers were acquired by getting the intersection of the differential metabolites from univariate statistics and multidimensional statistics between the NMDARE group and the control group, suggesting that the disparities between patients with greater homogeneity and the controls are amplified. In addition, seven differential metabolites were identified by the univariate statistics between the AE(TBA) group and the control group, including alpha‐linolenic acid and gamma‐linolenic acid, suggesting that the dysregulated biosynthesis of unsaturated fatty acids and alpha‐linolenic acid metabolism might be crucial in the AE(TBA) disease course.

The TCA cycle, alternately recognized as the citric acid cycle or the Krebs cycle, represents a sequence of reactions enclosed in a loop, establishing a metabolic powerhouse within cells. The TCA cycle serves as a central hub in cellular metabolism due to its capacity to receive multiple substrates. Pyruvate, the end product of glycolysis, holds paramount importance in various cell types, notably neurons. Within mitochondria, pyruvate undergoes oxidation facilitated by pyruvate dehydrogenase (PDH), resulting in the production of acetyl coenzyme A (acetyl‐CoA). This compound can subsequently bind with oxaloacetate (OAA) to initiate the formation of citrate, which serves as the primary substrate for the TCA cycle. Simultaneously, pyruvate may undergo carboxylation catalyzed by pyruvate carboxylase (PC) to yield oxaloacetate (OAA), which serves as a crucial anaplerotic pathway for replenishing intermediates of the TCA cycle. This process is not only vital for gluconeogenesis but also plays a significant role in other pathways, including the urea cycle and lipid synthesis [20]. So far, limited research has documented variations in pyruvate levels in CSF between patients with AE and the control subjects without neurological diseases. In this study, significant increases of pyruvic acid and oxoglutaric acid were identified in the AE(CBA‐panel) group compared to the control group, and they were also enriched in the TCA cycle, suggesting disordered cellular energy production and metabolism in the acute stage of AE. In an investigation into the acid–base balance of CSF among individuals with meningoencephalitis, it was observed that the level of pyruvic acid was most elevated in patients diagnosed with purulent meningoencephalitis, in contrast to those with lymphocytic meningitis and control subjects [21]. Furthermore, aberrant pyruvate metabolism contributes to the pathogenesis of several antibody‐mediated conditions, including multiple sclerosis (MS) [22], neuromyelitis optica [23], and systemic lupus erythematosus (SLE) [24]. McArdle and colleagues discovered an elevated level of pyruvate in individuals with MS [22]. Moreover, a heightened activity of pyruvate kinase was also observed in CSF of individuals with disseminated sclerosis [25], further reinforcing the association between disrupted pyruvate metabolism and the progression of MS. For a comparable assessment of metabolites across various brain regions in lupus‐prone MRL/lpr mice, a rise in pyruvate levels was noted in the frontal cortex but not in the hypothalamus. This implies that the frontal cortex might exhibit a greater susceptibility compared to the hypothalamus in SLE [24]. Consistent with other antibody‐mediated diseases, intermediates of the TCA cycle such as pyruvic acid and oxoglutaric acid were altered in the CSF from AE patients, suggesting disordered energy generation in the mitochondria. Except for the characteristics of pathologic processes in pyruvate, its potential therapeutic implications have also been investigated in certain neurological disorders. The application of pyruvate has demonstrated a significant reduction in infarct volume, enhancement in behavioral performance, and decrease in the number of neutrophils, microglia, and NFκB activation in rodent models following middle cerebral artery occlusion. The protective effects of pyruvate against ischemia are likely attributed to its anti‐inflammatory mechanisms [26]. However, due to the restricted sample size, we must regard the collection of potential CSF metabolite biomarkers as merely a tentative proposition. Significant changes might be made during a substantial increase in the number of patients per group.

Our targeted metabolomics analysis also identified valine, leucine, and isoleucine biosynthesis as another probable set of influential signaling pathways disrupted in the CSF of AE patients diagnosed by cell‐based assays. Ketoleucine (also known as α‐ketoisocaproic acid or KIC), derived from L‐leucine, is among the branched‐chain amino acids (BCAAs) implicated in energy metabolism [27, 28]. Prior experimental investigations have indicated that KIC induces dysfunction in mitochondrial bioenergetics [28], disrupts energy production [29], hampers mitochondrial pyruvate transport [30], and consequently leads to oxidative stress [31, 32] in the brains of rats. Indeed, neurons rely heavily on proper mitochondrial function for their normal operation [33]. Herein, the levels of KIC in the CSF of the AE(CBA‐panel) group were notably elevated compared to those in the control group, providing additional evidence of impaired mitochondrial function in the progression of advancement of AE. While no analogous findings regarding AE have been documented, changes in KIC levels have been observed in cases of MS. An integrated approach utilizing Q‐TOF LC/MS and LC–MS/MS for metabolomics investigation revealed reduced plasma KIC levels in pediatric multiple sclerosis patients compared to those in healthy controls [34]. In the Qiagen IPA enrichment analysis, KIC showed enrichment in central nervous system inflammation in patients with progressive form compared to healthy subjects [35]. Moreover, in uncovering the neuropathological mechanisms underlying the inherited neurometabolic disorder maple syrup urine disease, KIC modulates the phosphorylation of intermediate filaments in postnatal rat cortical slices via ionotropic glutamatergic receptors NMDA, AMPA, and kainate [36]. Together, our findings represent the initial evidence of KIC alteration in the CSF of AE patients, emphasizing the need for cautious interpretation and further validation.

In addition, one of the innovations of this study was to analyze the AE(CBA‐panel) group and the AE(TBA) group separately, because CBA‐ and TBA‐positive AE subtypes might represent distinct immunological and clinical subgroups. CBA is known to detect antibodies against specific neuronal surface antigens, often associated with a more acute clinical presentation and possibly distinct metabolic signatures due to their direct influence on neuronal signaling pathways [37, 38]. In contrast, TBA typically detects antibodies targeting intracellular antigens, which may trigger immune responses through different mechanisms, including cytotoxic T‐cell involvement, and often represent a chronic course with a different metabolic impact [39, 40, 41]. Thus, analyzing these groups separately allows us to identify subgroup‐specific metabolic disruptions that may otherwise be obscured in a combined analysis. Herein, we observed distinct metabolites that were dysregulated in each group when compared with the control group. In the AE(TBA) group, the prominent metabolites included methylcysteine, alpha‐linolenic acid, and gamma‐linolenic acid, among others, indicating disruptions in lipid metabolism and possibly chronic inflammatory responses [41, 42, 43, 44]. However, for the AE(CBA‐panel) group, except for the significant alterations of alpha‐linolenic acid and gamma‐linolenic acid, metabolites such as hydroxypropionic acid, phenylalanine, picolinic acid, and pyruvic acid were also altered. These metabolites are associated with neuronal metabolism, neurotransmission, and immune modulation, suggesting that surface antigen‐directed AE could impact pathways associated with neurotransmitter metabolism and oxidative stress [1, 45, 46, 47, 48, 49]. Collectively, differential metabolic disruptions in the AE(CBA‐panel) group and the AE(TBA) group could provide insights into subtype‐specific pathophysiological mechanisms, which might contribute to the identification of mechanism‐targeted therapeutic approaches in the future.

Despite successfully enrolling 18 patients diagnosed with AE and 17 controls without neurological disorders, the sample sizes for both cohorts remained relatively small, potentially limiting the robustness of our findings. To validate our results, it is essential to conduct metabolic analyses on CSF samples from larger and multicenter AE cohorts. Furthermore, the cohorts exhibited disparities in certain crucial parameters that were not aligned. For example, there was no significant difference in age between the NMDARE group and the control group, but significant age differences were observed between the AE(CBA‐panel) or the AE(TBA) groups and the control group. Thus, we advise readers to interpret our findings cautiously, and future studies should ensure proper matching of subjects for these critical parameters.

## 5 Conclusions

Collectively, different metabolic fingerprints of CSF were observed between the AE group and the control group, particularly involving metabolites related to mitochondrial dysfunction, which was beneficial in elucidating the pathophysiology of AE.

## Author Contributions

Xiaolong Li and Huihui Jiang contributed to study design, formal analysis, methodology, visualization, and original draft writing. Xiaolong Li, Xiaoxiao Qin, and Yuan Xie provided clinical patient data and CSF samples. Lingyun Wang, Jinwen Wang, Shushen Ji, and Huihui Jiang were involved with experimental operation, formal analysis, visualization, review, and editing. Huihui Jiang and Qun Wang conceived the study, conceptualization, data curation, review, and editing, project administration, resources, and supervision of the research.

## Ethics Statement

This study was approved by the Ethics Committee of the Xiangyang No. 1 People's Hospital, Hubei University of Medicine (Xiangyang, China). The corresponding Institutional Review Board (IRB) number was XYYYE20220075, in accordance with the Declaration of Helsinki. All participants provided written informed consent before inclusion in the study. Control CSF samples were collected from patients undergoing spinal anesthesia for elective non‐neurological surgical procedures. The risks and benefits were communicated to each patient, and the collection was performed only with explicit patient consent. The protocol for patient recruitment and sampling received approval from the ethics committee of the Xiangyang No.1 People's Hospital, Hubei University of Medicine.

## Conflicts of Interest

The authors declare no conflicts of interest.

## Supporting information


**FIGURE S1.** (A) A Venn diagram was utilized to illustrate the differential metabolites identified between the control group (*n* = 17) and the AE(CBA‐panel) group (*n* = 12), derived from both univariate and multidimensional statistical analyses.
**FIGURE S2.** Pathway enrichment analyses were conducted using the pathway‐associated metabolite sets (SMPDB) (A), the predicted metabolite sets (B), and the hsa set (C) for the control group (*n* = 17) and the AE(CBA‐panel) group (*n* = 12).
**FIGURE S3.** Pathway enrichment analyses were depicted based on the pathway‐associated metabolite sets (SMPDB) (A), the predicted metabolite sets (B), and the hsa set (C) for the control group (*n* = 17) and the NMDARE group (*n* = 5).
**FIGURE S4.** The arrangement of the heatmap displaying differential metabolites is categorized by class, with the names of individual samples presented for both the control group (*n* = 17) and the AE(TBA) group (*n* = 6).

**TABLE S1.** Clinical presentations of AE cases.

## Data Availability

The data that support the findings of this study are available on request from the corresponding author. The data are not publicly available due to privacy or ethical restrictions.
