# Supplementary material for: Metabolic Characterization of Cerebrospinal Fluid for Patients With Autoimmune Encephalitis: A Preliminary Study
Source: CNS Neurosci Ther. 2025 Jan 3;31(1):e70203. doi: 10.1111/cns.70203 (PMC11696248; doi:10.1111/cns.70203)
Supplement: Supplementary file 1 — FIGURE S1. (A) A Venn diagram was utilized to illustrate the differential metabolites identified between the control group (n = 17) and the AE(CBA‐panel) group (n = 12), derived from both univariate and multidimensional statistical analyses. FIGURE S2. Pathway enrichment analyses were conducted using the pathway‐associated metabolite sets (SMPDB) (A), the predicted metabolite sets (B), and the hsa set (C) for the control group (n = 17) and the AE(CBA‐panel) group (n = 12). FIGURE S3. Pathway enrichment analyses were depicted based on the pathway‐associated metabolite sets (SMPDB) (A), the predicted metabolite sets (B), and the hsa set (C) for the control group (n = 17) and the NMDARE group (n = 5). FIGURE S4. The arrangement of the heatmap displaying differential metabolites is categorized by class, with the names of individual samples presented for both the control group (n = 17) and the AE(TBA) group (n = 6). [file CNS-31-e70203-s001.docx]

Metabolic characterization of cerebrospinal fluid for patients with autoimmune encephalitis

**Xiaolong Li^1,2^** **Xiaoxiao Qin^1^** **Yuan Xie^2^ Lingyun Wang^3^ Jinwen Wang^3^ Shushen Ji^3^ Huihui Jiang^3^ Qun Wang^1,4,5^**

^1^Department of Neurology, Beijing Tiantan Hospital, Capital Medical University, Beijing, China

^2^Department of Neurology, Xiangyang No. 1 People's Hospital, Hubei University of Medicine, Xiangyang, Hubei Province, China

^3^Zhangjiang Center for Translational Medicine, Shanghai Biotecan Pharmaceuticals Co., Ltd., Shanghai, China

^4^National Center for Clinical Medicine of Neurological Diseases, Beijing, China ^5^Beijing Institute of Brain Disorders, Collaborative Innovation Center for Brain Disorders, Capital Medical University, Beijing, China

**Correspondence**

Qun Wang, 119 South Fourth Ring West Road, Fengtai District, Beijing 100070, China.

Email: wangq@ccmu.edu.cn

Huihui Jiang, 180 Zhangheng Road, Pudong New District, Shanghai 200135, China.

Email: hhjiang16@fudan.edu.cn

**Funding information**

The National Key R&D Program of China grant, Grant/Award Number: 2022YFC2503800; National Natural Science Foundation of China, Grant/Award Number: 82371449; Natural Science Foundation of Beijing Municipality, Grant/Award Number: 7232045, Z200024; Capital Health Research and Development of Special grants, Grant/Award Number: 2024-1-2041; Innovative Research Program of Xiangyang No.1 People's Hospital, Grant/Award Number: XYY2023MS08.


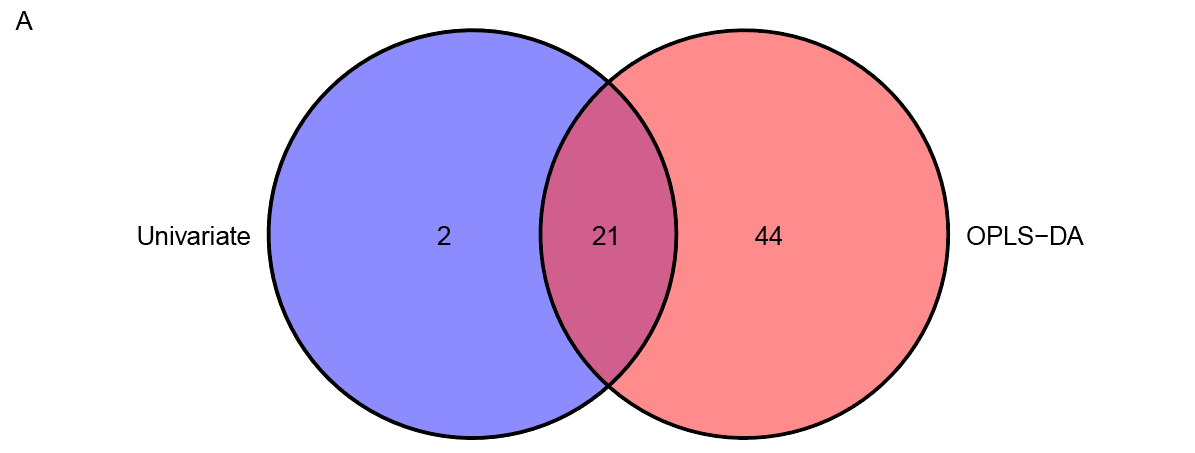

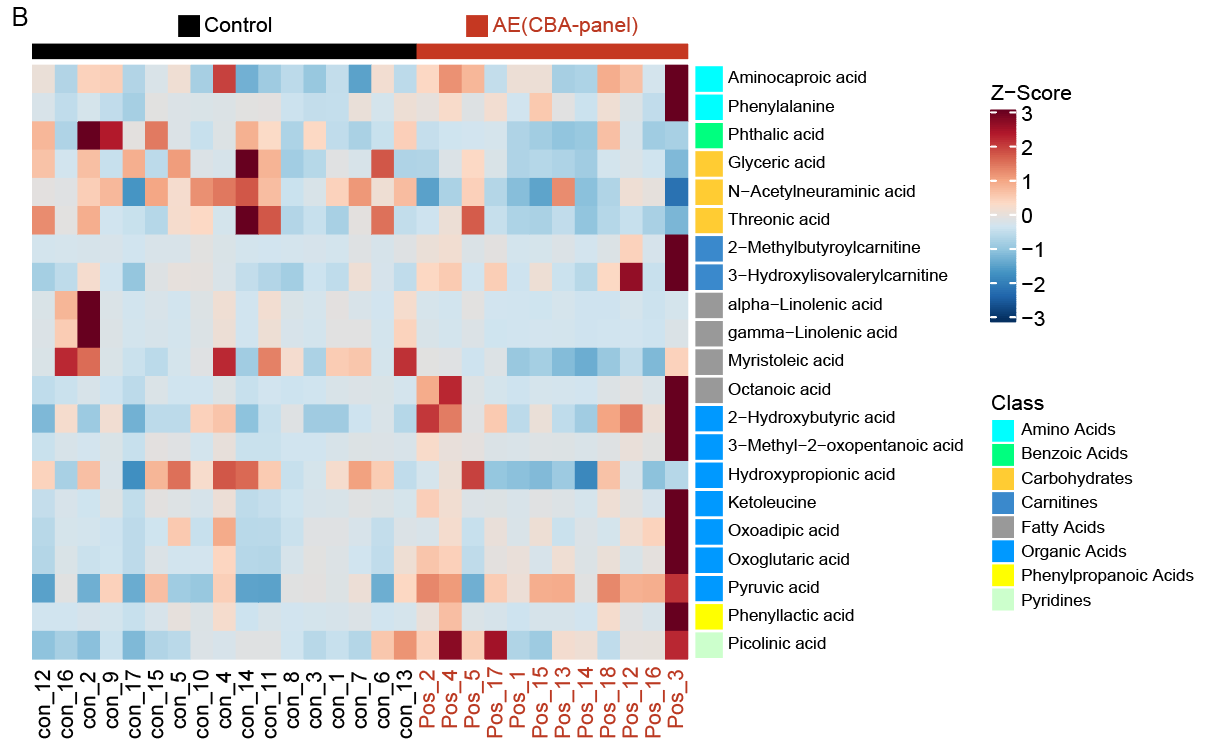


**SUPPLEMENTARY FIGURE** **1** (A) A Venn diagram was utilized to illustrate the differential metabolites identified between the control group (n=17) and the AE(CBA-panel) group (n=12), derived from both univariate and multi-dimensional statistical analyses. The differential metabolites in the intersection set were filtered by VIP > 1 in multi-dimensional statistics and P < 0.05 in univariate statistics. (B) The heatmap showcasing differential metabolites is arranged by class and includes the names of samples from these two groups.


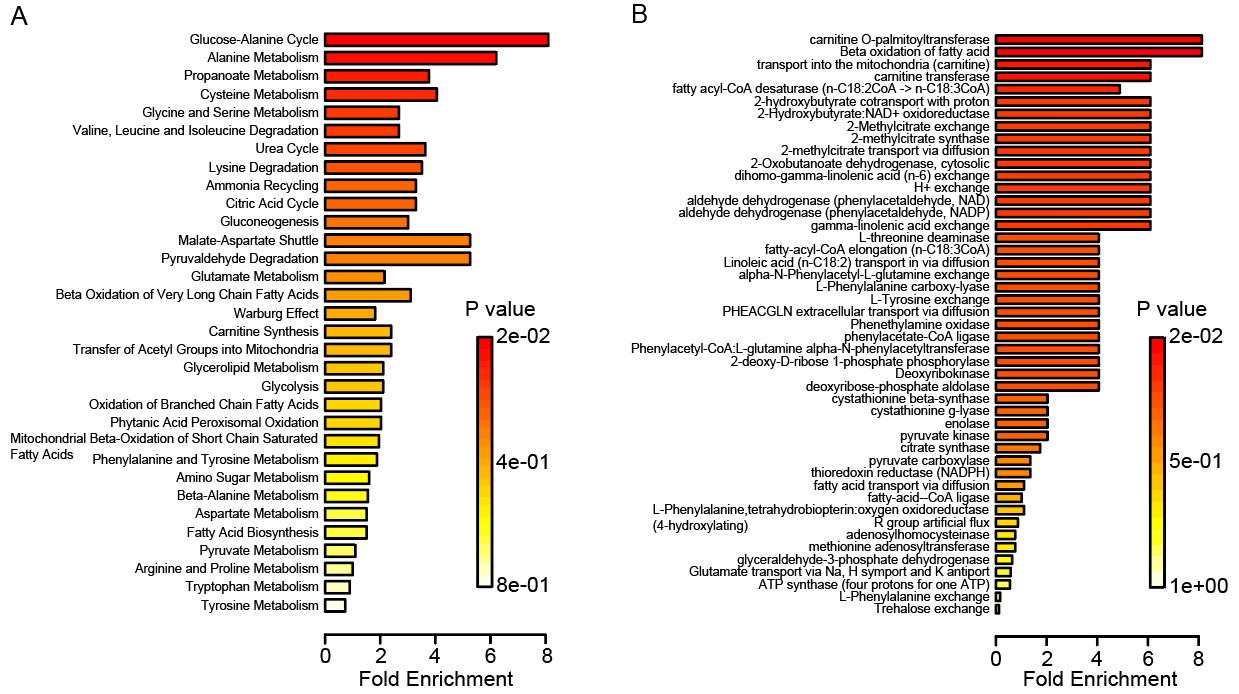

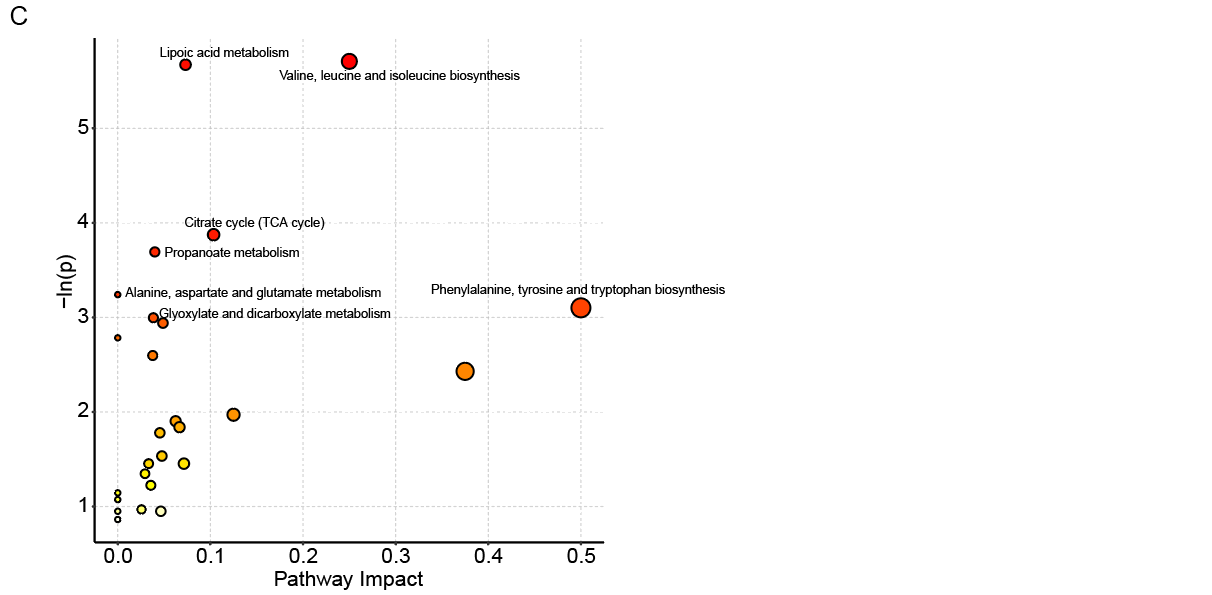


**SUPPLEMENTARY** **FIGURE 2** Pathway enrichment analyses were conducted using the pathway-associated metabolite sets (SMPDB) (A), the predicted metabolite sets (B), and the hsa set (C) for the control group (n=17) and the AE(CBA-panel) group (n=12).


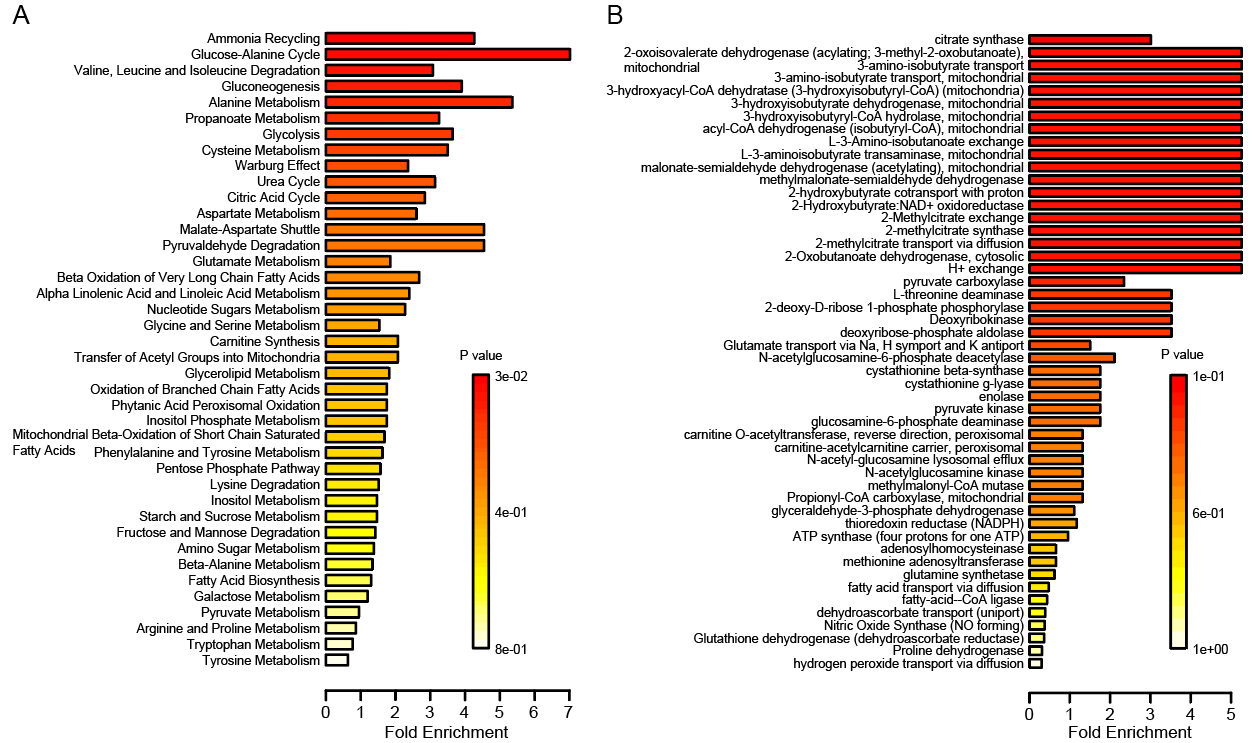

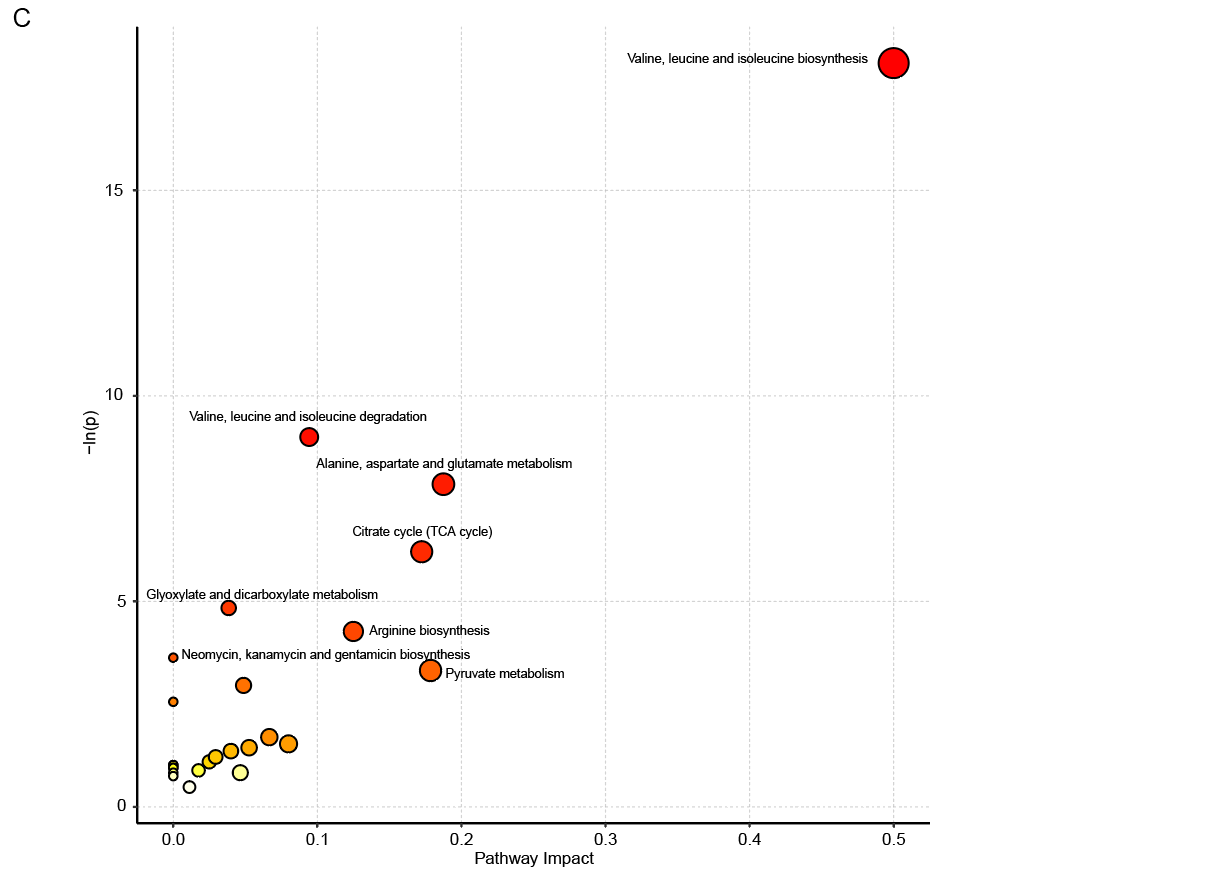


**SUPPLEMENTARY FIGURE 3** Pathway enrichment analyses were depicted based on the pathway-associated metabolite sets (SMPDB) (A), the predicted metabolite sets (B), and the hsa set (C) for the control group (n=17) and the NMDARE group (n=5).


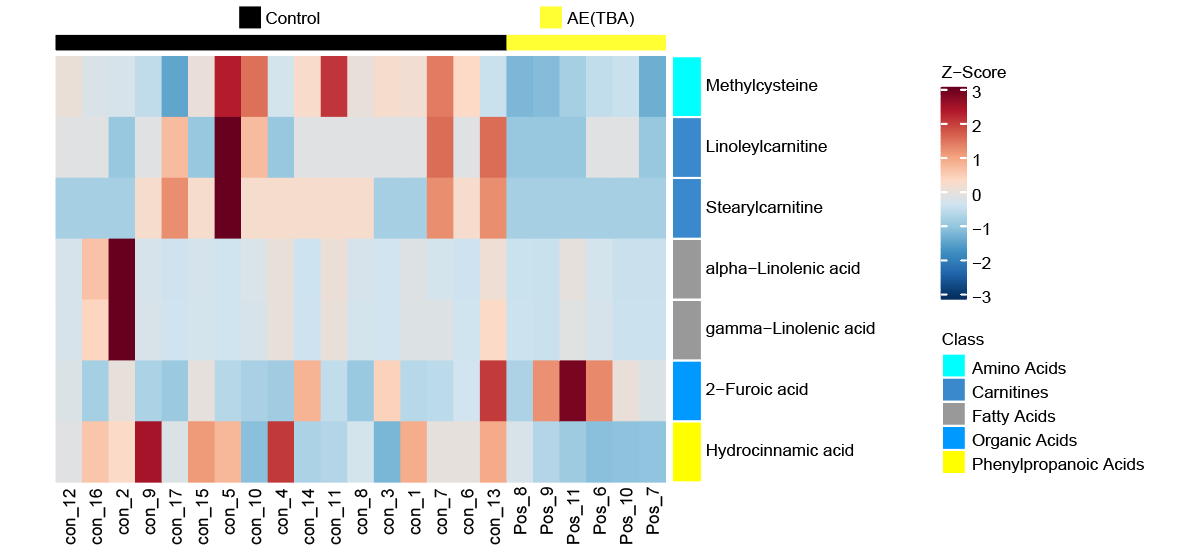


**SUPPLEMENTARY FIGURE 4** The arrangement of the heatmap displaying differential metabolites is categorized by class, with the names of individual samples presented for both the control group (n=17) and the AE(TBA) group (n=6).
